# Analysis of the transcriptional activity of genes of neuropeptides and their receptors in the blood of patients with thyroid pathology

**DOI:** 10.25122/jml-2020-0183

**Published:** 2021

**Authors:** Iryna Ivanivna Kamyshna, Larysa Borysivna Pavlovych, Vitaliy Antonovych Maslyanko, Aleksandr Mychailovich Kamyshnyi

**Affiliations:** 1. Department of Medical Rehabilitation, I. Horbachevsky Ternopil National Medical University, Ternopil, Ukraine; 2. Department of Clinical Immunology, Allergology and Endocrinology, HSEEU Bukovinian State Medical University, Chernivtsi, Ukraine; 3. Department of Microbiology, Virology, and Immunology, I. Horbachevsky Ternopil National Medical University, Ternopil, Ukraine

**Keywords:** neuropeptides, mRNA, autoimmune thyroiditis, hypothyroidism

## Abstract

The thyroid hormone plays a vital role in the development and maturation of the nervous system not only during prenatal and perinatal age but also in adults. “Peripheral marker hypothesis” revealed that gene expression changes in some regions of the brain are reflected into the peripheral blood lymphocytes. The objective of the study was to investigate changes in the gene expression profile of neuropeptides and their receptors in patients with different forms of thyroid pathology. One hundred fifty-three patients with thyroid pathology were enrolled in the study. They were divided into three groups: group 1 included 16 patients with postoperative hypothyroidism, group 2 included 65 patients with hypothyroidism resulting from autoimmune thyroiditis (AIT), and group 3 included 72 patients with AIT and elevated levels of anti-thyroglobulin (anti-Tg) and anti-thyroid peroxidase (anti-TPO) antibodies in the serum. We used a pathway-specific polymerase chain reaction (PCR) array (RT^2^ Profiler™ PCR Array Human Neurotrophins & Receptors, QIAGEN, Germany) to identify and verify neuropeptides and receptors pathway-focused gene expression in 12 individuals that were randomly selected from each group using real-time PCR. Our research identified that patients with postoperative hypothyroidism had a considerably increased expression of NPY1R, NTSR1, and NPY4R. The patients with hypothyroidism caused by autoimmune thyroiditis had considerably lower expression of NTSR1, while the expression of NPY1R increased. The mRNA levels of NPY2R and PNOC increased in the patients with elevated levels of autoantibodies anti-Tg and anti-TPO in the serum, and mRNA levels of NPY1R and NTSR1 decreased in this group of patients.

## Introduction

Even though that the effect of thyroid hormones on the brain is not in doubt, the mechanism of their effect on the brain of an adult is not fully understood [1]. It is known that the deficiency of thyroid hormones in the prenatal and perinatal periods is accompanied by severe cognitive impairment [2].

It has been revealed that L-thyroxine (T4) and L-3,5,3'-triiodothyronine (T3) are present in the adult human brain. The enzyme deiodinase type II, responsible for converting inactive T4 to active T3, is also present in glial and nerve cells [3].

Over a hundred different neuropeptides can be released from neurons and enter the bloodstream, which are reported to affect the function of immune cells [4]. At the same time, lymphocytes can be influenced by neurotransmitters in the blood or tissue. Neurotransmitters are also detected in the blood. For instance, the levels of substance P in the blood are elevated as a result of stroke [5]. In addition to being released by neurons, neurotransmitters and neurochemicals are also produced by other cells, mainly by lymphocytes [6], which may result in an autocrine and paracrine action on immune cells [7]. The expression of neurotransmitter receptors on lymphocytes permits brain signals to affect the immune system through the peripheral nervous system. In addition to exposure to lymphocytes, other immune cells have receptors for neurotransmitters and neurochemicals, pointing out that neural signaling regulates multiple aspects of the immune response [8].

We previously reported that autoimmune thyroiditis (AIT) and hypothyroidism could affect the expression of mRNA nerve impulse transmission genes in a gene-specific manner [9]. These changes in gene expressions can also play an important role in the development of neurological complications related to thyroid pathology. Taking into account that changes in the level of thyroid hormones affect the expression of such neurotrophins and regulators of synaptogenesis as glial cell line-derived neurotrophic factor (GDNF), brain-derived neurotrophic factor (BDNF), neurotrophin 3 (NTF3), cerebellin 1 precursor protein (Cbln1), we naturally assumed that the transcriptional profile of neuropeptides and their receptors in the blood in these groups of patients might also change.

It is still uncertain what is the exact mechanism of development of neurological complications in the dysfunction of thyroid glands. Also. it is not well-understood the mechanism that activates thyroid hormone-regulated gene expression in the adult brain. “Peripheral marker hypothesis” revealed that gene expression changes in some regions of the brain are reflected into the peripheral blood lymphocytes [10]. Transcriptome analysis is indicative of gene functional activity [11, 12]. In our study, we used polymerase chain reaction (PCR) arrays to compare gene expression data, which enables understanding of the influence of thyroid hormones and serum autoantibodies, such as an anti-thyroglobulin antibody (anti-Tg) and anti-thyroid peroxidase antibody (anti-TPO) on the mRNA neuropeptides and receptors pathway-focused gene expression in patients with primary hypothyroidism resulted from AIT and postoperative hypothyroidism and patients with AIT with rising serum autoantibodies, such as anti-Tg and anti-TPO.

## Material and Methods

One hundred fifty-three patients with thyroid pathology were enrolled in this study. They were divided into three groups: group 1 included 16 patients with postoperative hypothyroidism, group 2 included 65 patients with hypothyroidism resulting from AIT, and group 3 included 72 patients with AIT and elevated levels of anti-Tg and anti-TPO antibodies in the serum. The control group included 25 healthy individuals, who were recruited randomly, without age or sex matching. The clinical characteristics of the subjects are shown in Table 1.

**Table 1.** Clinical characteristics of the subjects.

	Control group (n=25)	Patients with postoperative hypothyroidism (Group 1) (n=16)	Patients with hypothyroidism as a result of AIT (Group 2) (n=65)	Patients with AIT and rising serum levels of anti-Tg and anti-TPO autoantibodies (Group 3) (n=72)
**Age (years)**	46.08±14.58	47.30±12.27	46.72±15.49	45.02±13.65
**fT4 (pmol/L)**	8.91±0.97	3.44±0.31	4.13±0.52	8.51±0.82
**TSH (mIU/mL)**	2.67±0.52	8.61±0.84	7.09±0.50	2.38±0.62
**anti-TPO (IU/mL)**	34.04±3.70	36.13±2.78	380.62±73.42	330.36±50.23
**anti-TG (IU/mL)**	15.32±1.97	15.50±1.90	32.97±4.27	36.38±7.70
**Current dose of L-thyroxine (μg/day)**	None	110.95±5.25	88.46±1.55	None

Data are expressed as mean ± standard deviation.

Hypothyroidism was diagnosed following the recommendations of the American Association of Clinical Endocrinologists (2012). The diagnosis of AIT was based on detected circulating antibodies to thyroid antigens (anti-TPO and anti-TG) and reduced echogenicity on thyroid ultrasound in a patient with relevant clinical features [13].

Blood specimens were collected between 8 and 10 AM after an overnight fast. Free thyroxine (fT4) (a normal range of 6.0–13.0 pmol/L for males and 7.0–13.5 pmol/L for females), thyroid-stimulating hormone (TSH) (a normal range of 0.3–4.0 mIU/mL), anti-TPO (a normal range of 0–30 IU/mL) and anti-TG (a normal range of 0–65 IU/mL) antibody levels were determined in every individual using the STAT FAX303/Plus analyzer (Awareness Technology Inc, USA).

Patients under the age of 18 or those suffering from malignancy, inflammation associated with rheumatic diseases or acute/chronic infection, diabetes mellitus, cardiovascular or cerebrovascular diseases, chronic hepatic or renal diseases, as well as pregnant women and those using any drugs that could interfere with thyroid function, were excluded from the study.

We used a pathway-specific PCR array (Neurotrophins & Receptors RT^2^ Profiler PCR Array, QIAGEN, Germany) to identify and verify neuropeptides and receptors pathway-focused gene expression in 12 individuals, which were randomly selected from each group using real-time PCR due to the procedure described below.

### Experimental procedures

#### RNA isolation

The total RNA was isolated from white blood cells using NucleoZOL (Macherey-Nagel, Germany), according to the manufacturer’s instructions. NucleoZOL is designed to isolate total RNA (small and large RNA) in single or separate fractions from a variety of sample materials, such as cells, tissue, and liquids of human or animal origin. White blood cells were lysed and homogenized in NucleoZOL reagent based on guanidinium thiocyanate and phenol.

#### cDNA synthesis

The RNA quality was determined, and it was reverse transcribed. The concentration and quality of the isolated total RNA were determined on a NanoDrop spectrophotometer (Thermo Scientific™, USA). For the reverse transcription procedure with a cDNA conversion RT^2^ First Strand Kit (QIAGEN, Germany, Cat. no. 330401), the RNA samples with the following parameters were selected: ratio A260/A280 within the range of 1.8–2.2.

The RT^2^ HT First Strand Kit procedure comprises two steps: elimination of genomic DNA contamination and reverse transcription, which enable fast and easy handling of 96 RNA samples simultaneously. After genomic DNA elimination, the RNA sample undergoes reverse transcription with an RT master mix, as well as random hexamers and oligo-dT prime reverse transcription to capture more difficult-to-detect genes.

#### PCR Array

The cDNA was then handled with the RTI Profiler PCR Array (QIAGEN, Cat. no. PAHS-031Z) in combination with the RTI SYBR® Green qPCR Mastermix (QIAGEN, Cat. no. 330504), following the complete RT^2^ Profiler PCR Array procedure (www.qiagen.com). Samples were assigned to control and study groups. Cycle threshold (CT) values were normalized based on an automatic selection from the full panel of reference genes. Any CT value >35 was considered to be a negative call. The RT^2^ Profiler PCR Array data analysis software calculates the fold change based on the widely used and agreed upon the delta-delta CT(ΔΔCT) method. The data analysis web portal calculates the fold change/regulation using the delta-delta CT method, in which delta CT is calculated between the gene of interest (GOI) and an average of reference/housekeeping genes (HKG), followed by delta-delta CT calculations (delta CT (Test Group)-delta CT (Control Group)). Fold Change is then calculated using the 2^ (-delta-delta CT) formula. This data analysis report was exported from the QIAGEN web portal at GeneGlobe. The software allows defining the best reference genes for normalization. Also, it allows defining the best reference genes for normalization. In further analysis, neuropeptides and receptor pathway-focused genes were selected for this work; a list of these genes is given in Table 2.

**Table 2.** Neuropeptides and receptor pathway-focused gene expression analysis.

Unigene	Refseq	Symbol	Description
**Hs.129**	NM_000730	CCKAR	Cholecystokinin A receptor
**Hs.75294**	NM_000756	CRH	Corticotropin-releasing hormone
**Hs.567282**	NM_005314	GRPR	Gastrin-releasing peptide receptor
**Hs.158348**	NM_001524	HCRT	Hypocretin (orexin) neuropeptide precursor
**Hs.248144**	NM_000529	MC2R	Melanocortin 2 receptor (adrenocorticotropic hormone)
**Hs.733076**	NM_003717	NPFF	Neuropeptide FF-amide peptide precursor
**Hs.99231**	NM_053036	NPFFR2	Neuropeptide FF receptor 2
**Hs.1832**	NM_000905	NPY	Neuropeptide Y
**Hs.519057**	NM_000909	NPY1R	Neuropeptide Y receptor Y1
**Hs.37125**	NM_000910	NPY2R	Neuropeptide Y receptor Y2
**Hs.590869**	NM_002531	NTSR1	Neurotensin receptor 1 (high affinity)
**Hs.88218**	NM_006228	PNOC	Prepronociceptin
**Hs.524719**	NM_005972	NPY4R	Pancreatic polypeptide receptor 1
**Hs.633301**	NM_001058	TACR1	Tachykinin receptor 1
**Hs.534363**	NM_003353	UCN	Urocortin

#### Statistical analysis of PCR array data

The RT^2^ Profiler PCR Array Data Analysis software does not perform any statistical analysis beyond the calculation of p-values using a Student’s t-test (two-tail distribution and equal variances between the two samples) based on the triplicate 2^(-ΔCT) values for each gene in the experimental group compared to the control group. The Microarray Quality Control (MAQC) published results indicating that a ranked list of genes based on a fold-change and such a p-value calculation was sufficient to demonstrate reproducible results across multiple microarrays and PCR Arrays, including the RT^2^ Profiler PCR Arrays.

## Results

The results from the RT^2^ Profiler Neuropeptides and receptor pathway-focused gene expression analysis indicated that in Group 1, which includes patients with postoperative hypothyroidism, among anti-apoptotic genes, the expression of NPY1R and NTSR1 was increased by 3.0 and 3.7 times, respectively. At the same time, the expression of CCKAR, CRH, GRPR, HCRT, MC2R, NPFF, NPFFR2, NPY, NPY2R, PNOC, TACR1, and UCN did not change in this group of patients, as was shown in Table 3.

**Table 3.** Differential expression of mRNA neuropeptides and receptor pathway-focused genes in patients with different thyroid pathologies.

Gene Symbol	Up-Down Regulation (comparing to the control group)		
	Patients with postoperative hypothyroidism (Group 1)	Patients with hypothyroidism as a result of AIT (Group 2)	Patients with AIT and rising serum anti-Tg and anti-TPO autoantibodies (Group 3)
	Fold Regulation	Fold Regulation	Fold Regulation
**CCKAR**	-1.2 (p=0.13)	-1.01 (p=0.97)	-1.07 (p=0.62)
**CRH**	-1.05 (p=0.2)	-1.05 (p=0.43)	-1.08 (p=0.06)
**GRPR**	-1.08 (p=0.63)	1.06 (p=0.81)	-1.15 (p=0.49)
**HCRT**	-1.05 (p=0.2)	-1.05 (p=0.43)	-1.08 (p=0.06)
**MC2R**	-1.05 (p=0.2)	-1.05 (p=0.43)	-1.08 (p=0.06)
**NPFF**	-1.05 (p=0.2)	-1.05 (p=0.43)	-1.08 (p=0.06)
**NPFFR2**	-1.17 (p=0.86)	1.05 (p=0.53)	-1.19 (p=0.12)
**NPY**	-1.16 (p=0.27)	-1.06 (p=0.82)	-1.21 (p=0.12)
**NPY1R**	3.0 (p=0.05)	5.5 (p=0.0006)	-3.16 (p=0.003)
**NPY2R**	-1.05 (p=0.2)	-1.05 (p=0.43)	4.63 (p=0.06)
**NTSR1**	3.73 (p=0.03)	-3.71 (p=0.007)	-3.56 (p=0.005)
**PNOC**	1.18 (p=0.27)	1.09 (p=0.62)	4.63 (p=0.004)
**NPY4R**	3.21 (p<0.05)	-1.01 (p=0.95)	1.01 (p=0.97)
**TACR1**	-1.06 (p=0.61)	-1.17 (p=0.34)	-1.13 (p=0.61)
**UCN**	1.02 (p=0.98)	-1.06 (p=0.92)	-1.21 (p=0.43)

The p values are calculated based on a Student's t-test of the replicate 2^(- Delta CT) values for each gene in the control group and study groups.

**Figure 1.**
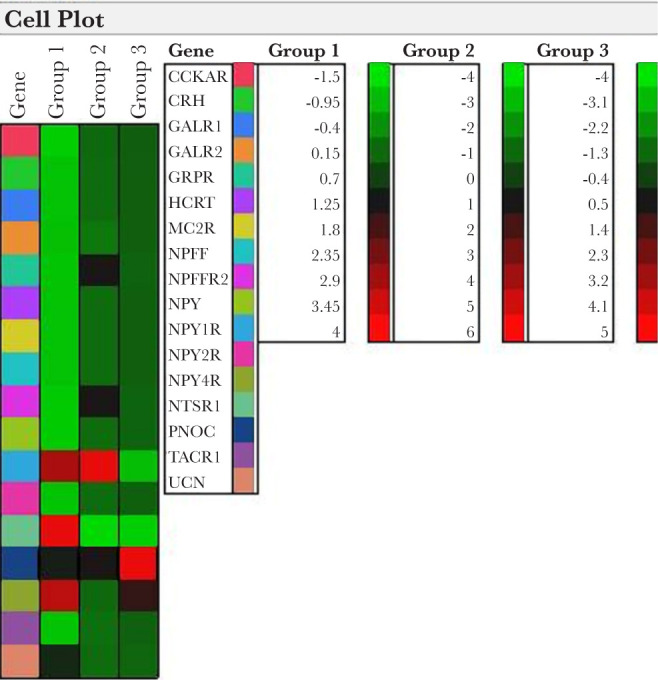
Cell plot neuropeptides and receptor pathway-focused gene expression analysis.

In patients with hypothyroidism as a result of AIT (Group 2), the expression of NPY1R was upregulated (5.5-fold). On the other hand, NTSR1 was downregulated by 3.7 times (Figure 1). As shown in Table 2, the expression of CCKAR, CRH, GRPR, HCRT, MC2R, NPFF, NPFFR2, NPY, NPY2R, PNOC, TACR1, and UCN did not change in this group.

We noted that in Group 3, which includes patients with AIT and rising serum anti-Tg and anti-TPO autoantibodies, NPY1R was down-regulated by 3.16 times. Simultaneously, the mRNA level of NPY2R was significantly increased (4.6-fold) (Figure 1). Reduction in NTSR1 (3.6-fold) mRNA was found in Group 3. The expression of PNOC was markedly increased in Group 3 (4.6-fold). Meanwhile, in a group of patients with postoperative hypothyroidism, the expression of CCKAR, CRH, GRPR, HCRT, MC2R, NPFF, NPFFR2, NPY, NPY2R, TACR1, and UCN did not change.

## Discussion

AIT is one of the most common autoimmune diseases associated with immunogenicity [14]. Neuropeptide Y (NPY), which belongs to the NPY family, is expressed in both the central and peripheral nervous systems [15]. Thyroid hormones have been shown to regulate the release of NPY [16]. In our study, we found no changes in NPY transcriptional activity in all groups of patients.

Since the NPY mRNA level in patients did not change, we can assume that the mechanisms of influence of AIT and hypothyroidism, which lead to changes in the composition of neuropeptides, may not involve direct gene transcription, but TR mediates it. Moreover, the expression of various Y receptors on immune cells enables NPY to influence immune function directly [17].

Thus, one of the most widespread receptors, which is found in all immune cells, is the Y1 receptor [18]. According to the literature, the expression of genes for Y1 and Y2 receptors in mice was detected in macrophages [19]. Other studies support the expression of Y1 and Y2 receptors in rat granulocytes [20].

We found that in patients with hypothyroidism related to AIT and postoperative hypothyroidism, NPY1R expression increased. At the same time, significant suppression of NPY1R in the group of AIT patients with an increase in the serum level of autoantibodies such as anti-Tg and anti-TPO was observed.

Besides, we did not observe any changes in the NPY2R expression in patients with AIT-related hypothyroidism along with postoperative hypothyroidism. On the contrary, NPY2R was considerably increased in a group of patients with rising serum autoantibodies. Based on these data, we can assume that the high level of serum autoantibodies, such as anti-Tg and anti-TPO, will up-regulate the expression of NPY2R.

In our study, the expression of the NPY4R gene that encodes the Y4 receptor is increased in patients with postoperative hypothyroidism. At the same time, we did not find changes in the transcriptional activity of the NPY4R gene in other patient groups. Four human receptors of the NPY family are expressed in the hypothalamic regions of the brain that regulate appetite and energy metabolism [21], as well as in the periphery [22].

The absence of changes in the expression of NPY4R in patients with AIT-related hypothyroidism and a group of patients with rising serum autoantibodies suggests that the high level of serum autoantibodies, such as anti-Tg and anti-TPO, do not affect the expression of NPY4R.

A significant decrease in the expression of CRH mRNA in the paraventricular nucleus (PVN) of the hypothalamus in rats with hypothyroidism was revealed [23].

According to the literature, thyroid hormones affect various components of the hypothalamus-pituitary-adrenal (HPA) axis [24]. Thus, it has been shown that experimentally induced hypothyroidism can reduce the weight of the adrenal glands [25].

According to another study, there is an increase in the CRH gene transcription in the PVN of the hypothalamus after thyroidectomy.

Thyroidectomy can also contribute to increased transcription of the CRH gene in the PVN of the hypothalamus [26]. At the same time, our investigation found no changes in the expression of CRH in all groups of patients.

One of the most studied neurotensin (NT) receptors is NTSR1, which is widespread in the central nervous system. Its expression is observed in neurons, as well as in glial cells [27]. The involvement of NT in the regulation of thyroid hormone activity has been described previously [28]. On the other hand, the role of NT in the occurrence of mental and other diseases of the central nervous system has been studied [29]. In our research, we also found a change in the mRNA expression of NTSR1. So, there was a decrease in the expression of NTSR1 both in the patients with increased levels of anti-Tg and anti-TPO autoantibodies in the serum and patients with hypothyroidism caused by AIT compared to the increased NTSR1 expression in the patients with postoperative hypothyroidism.

When examining the expression of prepronociceptin (PNOC), a gene encoding a protein, we found an increase in the expression of this gene in the patients with elevated levels of anti-Tg and anti-TPO autoantibodies in the serum while in other groups was not changed.

We found no changes in the transcriptional activity of several genes. The expression of CCKAR, GRPR, HCRT, MC2R, NPFF, NPFFR2, TACR1, and UCN was not altered in any of the patient groups. Changes in their transcriptional profile are more considerable in the nervous tissue or tissue of the thyroid gland. Neuropeptides can influence the nervous system by modulating neurotransmitters’ release and action, functioning as trophic factors, or up-regulating the synthesis of neurotransmitter receptors [30].

## Conclusions

Our research found that patients with postoperative hypothyroidism had a considerably increased expression of NPY1R, NTSR1, and NPY4R. The expression of NTSR1 was significantly lower compared to the increased expression of NPY1R in patients with hypothyroidism caused by autoimmune thyroiditis. The mRNA levels of NPY2R and PNOC increased in the patients with elevated levels of anti-Tg and anti-TPO autoantibodies in the serum. Moreover, mRNA levels of NPY1R and NTSR1 decreased in this group of patients. Simultaneously, many genes, like CCKAR, CRH, GRPR, HCRT, MC2R, NPFF, NPFFR2, NPY, TACR1, and UCN, did not change their expression.

Nowadays, many of these points are still pressing but were not studied adequately; thus, further studies are required to resolve this issue. The effect of thyroid hormones on the brain is beyond dispute since hormone insufficiency may cause various neurological complications. Early diagnosis of nervous system damage associated with the diseases of the thyroid gland is of great importance for prevention and treatment. In this study, we have emphasized the significant role of neurotransmitters in the pathogenesis of thyroid pathology and showed both the importance of neurotransmitters in regulating immunity and the potential to enhance neurotransmitter activity for therapy.

## Acknowledgments

### Ethical approval

The approval for this study was obtained from the Ethics Committee of the HSEEU “Bukovinian State Medical University” and Chernivtsi Regional Endocrinology Center, Ukraine (approval ID: 11-07.11.2017).

Our study was conducted according to the Declaration of Helsinki adopted in 1975 and revised in 2008, and the ethical principles were entirely respected.

### Consent to participate

Written informed consent was obtained from the participants.

### Data availability

The data of this study is available by request.

### Conflict of interest

The authors declare that there is no conflict of interest.
